# Feasibility of Utilizing Recycled Aggregate Concrete for Revetment Construction of the Lower Yellow River

**DOI:** 10.3390/ma12244237

**Published:** 2019-12-17

**Authors:** Pan Feng, Honglei Chang, Guodong Xu, Qiaoling Liu, Zuquan Jin, Jian Liu

**Affiliations:** 1 School of Material Science and Engineering, Southeast University, Nanjing 211189, China; pan.feng@seu.edu.cn; 2 School of Qilu Transportation, Shandong University, Jinan 250002, China; 3 Jiangsu Testing Center for Quality of Construction Engineering, Nanjing 210028, China; seuxgd@126.com; 4 School of Civil Engineering, Shandong Jianzhu University, Jinan 250101, China; lql263@sdjzu.edu.cn; 5 School of Civil Engineering, Qingdao University of Technology, Qingdao 26033, China; jinzuquan@126.com

**Keywords:** revetment engineering, recycled aggregate, concrete, mechanical properties, freeze-thawing, carbonation

## Abstract

To explore the feasibility of utilizing recycled aggregate concrete (RAC) in revetment construction of the lower Yellow River, a series of mix proportions with local recycled aggregates (RA) were designed to evaluate its mechanical properties and durability. The morphology and micro-hardness of the interface transition zone (ITZ) were also characterized to explain the performance of RAC. Based on the compressive strength data of 13 groups of mixtures, which is larger than 30 MPa, and with the RA substitution rate not less than 50%, the RAC containing 50% recycled fine aggregate (RFA) (HDX50), 70% RFA (HDX70), and 50% recycled coarse aggregate (RCA) (HDC50) were selected. The experiment results suggest that the mechanical performance, frost resistance, and carbonation resistance of the selected RAC is generally poorer than that of natural aggregate concrete (NAC), but can meet the performance requirement of concrete for the revetment construction of the lower Yellow River. The comprehensive performance of these three mixtures ranks as: HDX50 > HDX70 > HDC50. When considering the RA substitution ratio as a priority, HDX70 would be the best choice and can be applied in the revetment engineering. A number of defects are observed on the surface of RA with old pastes attached. Furthermore, the ITZs formed around RA are loose and with low micro-hardness, which is deemed to be the dominating reasons leading to the weaker performance of RAC than that of NAC.

## 1. Introduction

The Yellow River, the second largest river in China, has nurtured generations of Chinese people, but its river flood has also caused great losses to human’s lives and properties for thousands of years. In recent decades, the Yellow River conducted integrated management, and the construction of revetment was one of the most important steps [1,2] because it protected the earthy levee from the damage of water flow, waves, or ice covers. The revetment is now mainly constructed by natural stone masonry and works well. However, large natural stone exploitation has caused extensive impact on environment, and hence is restricted. Consequently, the revetment construction is being suspended or postponed. Therefore, other replacement material is urgently needed to guarantee the timely construction of the revetment.

Concrete is believed to be a promising replacement, considering that it is a cheap construction material with excellent properties, and it has been successfully used in some river management projects. The biggest problem is that the aggregates needed for concrete, such as gravels and river sands, are also in shortage, and their exploitation will cause damage to the environment like natural stone masonry. Replacing natural aggregates (NAs) with recycled aggregates (RAs) to produce recycled aggregate concrete (RAC) blocks provides an eco-friendly and cost-effective alternative, especially given that tons of waste concrete are produced every year in cities along the Yellow River due to the demolition of old buildings [3,4].

RAC has been studied for several decades, and many researches [5,6,7,8,9] focus on its mechanical properties and durability. Ravindrarajah et al. [5] conducted one of the earliest experiments with recycled fine aggregate (RFA), and the results showed that the compressive and splitting tensile strength reduced slightly and the elasticity modulus decreased by about 15% when natural fine aggregate was totally replaced by RFA. However, when the substitution rate of RFA is less than 30%, several studies [10,11,12,13,14,15] found that the mechanical properties of RAC were limitedly influenced. Furthermore, Xiao et al. [16] reported a remarkable reduction in compressive strength of RAC, especially when the natural coarse aggregate (NCA) was 50% replaced by recycled coarse aggregate (RCA). Besides, Silva et al. [6] found that the elastic modulus of RAC decreased with increasing RCA. In general, the mechanical properties of RAC are worse than that of natural aggregate concrete (NAC), especially when the RA replacement is large. This could be attributed to the defects of RA, the weak interface between aggregate and paste, and the higher water absorption of the adhered mortar on RCA [6,7,8,9,17,18,19,20].

The frost resistance, carbonation resistance, chloride penetration resistance, and impermeability are often used to evaluate the durability of concrete. Generally, RAC is not recommended to use in harsh conditions [7] because of its weak frost resistance. Haitao [20] reported that the compressive strength, flexural strength, and splitting tensile strength of RAC decreased when the freezing and thawing cycles increased. Similar phenomena were reported by Salih [21], Yue [22], and Chen [23] that the relative elastic modulus decreased and the mass loss of concrete increased when exposed to freezing and thawing cycles. In addition, Wu [24] discovered that, with the increase of freeze-thawing cycles, both the compressive strength and modulus of RAC decreased linearly, and its compressive strength was lower than that of NAC after the same cycles. To improve the frost resistance of RAC, Salem et al. [25] infused 5% gas content and made RAC suitable for moderately cold climates.

Similar to the mechanical properties and frost resistance, the carbonation resistance of RAC usually decreases with the increase of RA dosage [7,26,27]. Silva et al. [27] found that the carbonation depth of RAC with 100% RCA replacement became 2.5 times larger than the reference. This value increased to 8.7 times when the aggregates were 100% replaced by RFA, probably due to the higher water absorption rate of RFA. However, according to the study of Lei [26], the carbonation depth of RAC could be lower than that of NAC under the same carbonation condition when the substitution ratio of RCA is over 70% and the adhered mortar content of RCA exceeds 40%. The authors attributed these seemingly abnormal results to the higher content of adhered mortar, which actually increases the total cement content and slows down the carbonation. In summary, the impact of RA on carbonation resistance of RAC is very complicated owning to the variations in material properties.

Furthermore, according to the previous investigations [28,29,30], the impermeability of RAC decreased with the increase of the RA content. Additionally, the chloride penetration resistance of RAC was generally lower than that of NAC [31,32,33]. However, it has been found that when RAC is prepared at a low water to cement ratio (W/C), it exhibits better performance than NAC in chloride attack environments, probably because of the higher C-S-H gel content in RAC, which enhances the chloride binding capacity [34,35,36].

Based on the above, it can be found that there have been a number of studies about RAC, and almost all of them indicate that the properties of RAC are generally poorer than that of NAC. A few contradictory findings are mainly caused by the different material properties of RA, which can be further traced back to the sources and locations, mix proportions, impurities, such as red block residues and soil, and the service history. All the differences mentioned above make the microstructure of the interface transition zones (ITZ) between the RA and the new cement paste and the RA and the old mortar differ a lot and are very complicated [37,38], which greatly influences the performance of RAC.

RAC has been applied to many recent projects [39,40], such as road constructions and non-load bearing structures. However, the application of RAC on revetment constructions has rarely been reported. Particularly, the revetment construction of the lower Yellow River has high requirements for the performance of RAC. On the one hand, the RAC blocks are required to have high mechanical performances (compressive strength >30 MPa and flexural strength >4 MPa) to resist the impact load of the water stream, storm, and ice cover. On the other hand, RAC blocks need to have sound frost resistance (the temperature of the Yellow River is lower than 0 °C for about 60 days per year) to withstand the freezing and thawing cycles.

## 2. Experiments

### 2.1. Raw Materials 

The raw materials used in this study consisted of cement, NA, RA, water, and water reducer. The cement was PO 42.5 (Portland Cement), produced by Shanshui Cement Co., Ltd. (Jinan, China), and its chemical composition is shown in Table 1. The natural coarse aggregates (NCAs) were gravels with good grading and the maximum size was less than 25 mm. The natural fine aggregates (NFAs) were river sand with 3.0 fineness modulus (medium sand). The water reducer was the polycarboxylate superplasticizer produced by Qinfen Building Material Company (Jinan, China).

**Figure 1:**
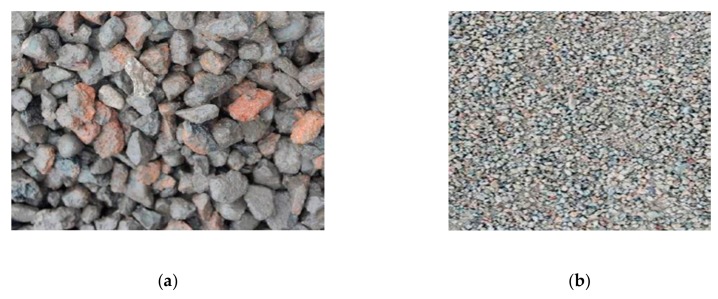
Macroscopic appearance of recycled aggregates: (**a**) Coarse aggregates (**b**) fine aggregates.

RA was from the derelict buildings in Jinan city, Shandong province, and had been processed by Shandong Mingran Renewable Resources Utilization Co., Ltd. The processing was mainly about removing the steel bars and most of the red bricks. The appearance of RA is shown in Figure 1. It was found that RAC was mainly made up of old paste attached to old graves and red bricks, while RAF mainly consisted of broken old pastes and red bricks. Moreover, the particle size of RCA was in the range of 5–25 mm with good grading, and that of RFA was less than 4.75 mm with 2.7 fineness modulus (medium sand). The properties of RA are presented in Table 2.

### 2.2. Concrete Mix Proportions

Three groups of RAC were designed: single mixed with RCA, single mixed with RFA, and double mixed with RCA and RFA. Each series includes 4 mix proportions: The substitution ratio of RA being 30%, 50%, 70%, and 100% of NA by mass, respectively. The label of all mixes and the detailed information are shown in Table 3.

### 2.3. Casting and Curing

After stirring, concrete paste was poured into molds of different sizes. Specimens for compressive strength and carbonation resistance tests were cast in molds with a size of 100 mm × 100 mm × 100 mm, while specimens for flexural strength and frost resistance tests were cast in molds with a size of 100 mm × 100 mm × 400 mm. After casting, the mold surfaces were sealed with thin film to prevent moisture evaporation. Then specimens were demolded after 24 h and cured at 23 ± 1 °C, at relative humidity of 95%. Specimens for compressive strength were cured for 3 days, 7 days, 28 days, 56 days, and 90 days separately, and all other specimens were cured for 28 days.

Note that the slump of all mixtures in casting falls in the range of 150~180 mm. The slump of RAC is slightly lower than that of NAC with the same dosage of water reducer. Additionally, the slump of RFA concrete approximates that of RCA concrete with different RA admixtures, attributed to the fact that the water absorptivity of the former is higher, while the actual dosage of the latter is greater. The slump of concrete with mixed RFA and RFC is a little lower. Therefore, when their dosage is higher than 50%, more water reducer is added to reach similar workability to that of concrete with only RFA or RFC, which aims to alleviate the impact of workability on the performance of different mixtures as much as possible.

### 2.4. Compressive Strength and Flexural Strength

The compressive strength and flexural strength were tested abiding by the GB/T 50081-2002 standard for test method of mechanical properties on ordinary concrete [41]. As introduced in Section 2.3, specimens of size 100 mm × 100 mm × 100 mm and 100 mm × 100 mm × 400 mm were prepared for compressive strength tests and flexural strength, using 200 t compressive strength tester and 10 t flexural strength tester with the loading 0.5 MPa/s and 0.05 MPa/s, separately. Three specimens of each mix were randomly chosen, and the average value of them was used for analysis.

### 2.5. Freezing and Thawing 

According to the DG/TJ 08-2018-2007 technical code on the application of recycled aggregate concrete [42], the frost resistance requirements for RAC are presented in Table 4. The RAC investigated in this study is for revetment construction of the lower Yellow River, which belongs to the “Positions impacted by cyclic wetting-drying or water level changing”, and the frost resistance grade should be over F50. Therefore, 75 freezing and thawing cycles were adopted to evaluate the frost resistance of RAC specimens.

**Figure 2:**
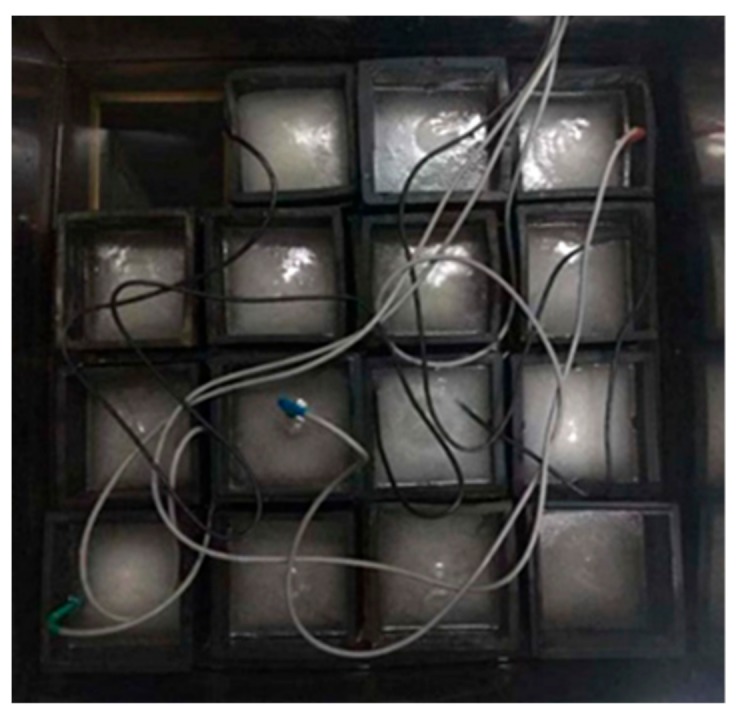
Freezing and thawing testing.

The frost resistance test was conducted abiding by the GB/T 50082-2009 standard for test methods of long-term performance and durability of ordinary concrete [43]. Specimens with a size of 100 mm × 100 mm × 400 mm were used. The core temperature of specimens was kept at (−18 ± 2) °C and (5 ± 2) °C during freezing and thawing. The water surface in the container was at least 5 mm above the specimen surface. Relative dynamic modulus and mass loss were measured for specimens subjected to freezing and thawing for 0, 25, 50, and 75 cycles, respectively, with the use of Equations (1) and (2). The average value of three specimens was adopted. The lab photo of the frost resistance test is shown in Figure 2.

(1) $$P_{i}=\frac{f_{ni}^{2}}{f_{oi}^{2}}\times 100$$

where, *P_i_* is the relative dynamic modulus of specimens after *n* freezing and thawing cycles; *f_ni_* is the dynamic modulus of specimens after *n* freezing and thawing cycles, *Hz*; *f_oi_* is the original dynamic modulus of specimens before the test, *Hz*.

(2) $$\Delta W_{\mathrm{ni}}=\frac{W_{\mathrm{oi}}-W_{\mathrm{ni}}}{W_{\mathrm{oi}}}\times 100$$

where, *ΔW_ni_* is the mass loss rates of specimens after *n* freezing and thawing cycles, %; *W_ni_* is the mass of concrete after *n* freezing and thawing cycles, g; *W_oi_* is the original mass of concrete before the test, g.

### 2.6. Carbonation 

**Figure 3:**
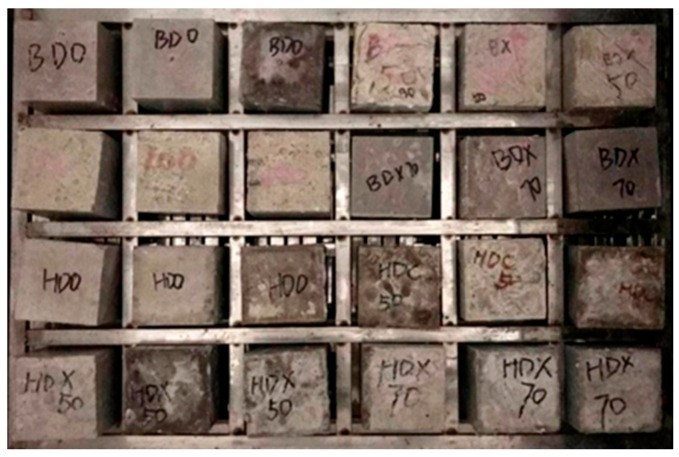
Carbonation testing.

The carbonation resistance test was conducted abiding by the GB/T 50082-2009 standard for test methods of long-term performance and durability of ordinary concrete [43]. Cubic specimens (100 mm × 100 mm × 100 mm) were first dried in an oven at 60 °C for 3 d; then five surfaces of each specimen were sealed with paraffin. The specimens were placed in an accelerated carbonation chamber with the CO_2_ concentration at (20 ± 1)%, relative humidity at (70 ± 5)%, and the temperature at (23 ± 2) °C. It should be noted that the distance between specimens should be larger than 50 mm to guarantee all the specimens have the same boundary conditions. The lab photo of the carbonation resistance test is presented in Figure 3. The carbonation depths were measured after 3 days, 7 days, 14 days, and 28 days of exposure, based on which carbonation rates were calculated.

The carbonation depth was tested with the method below. We sprayed phenolphthalein of 1% concentration on the split surface. Due to the alkaline solution of concrete before carbonation, the uncarbonated area turned to purple, whereas the carbonated area stayed unchanged. We marked 12 equally spaced test points along the boundary between the purple area and unchanged color area and measured the distance between each test point and the end surface of the unchanged color area. The carbonation depth was obtained by averaging the 12 measured distances. The carbonation time and depth was reported to follow the empirical relation described in Equation (3), by which the carbonation rate *k* can be calculated.

(3) $$x=k\sqrt{t}$$

where, *x* is the carbonation depth, mm; *k* is the carbonation rate; *t* is the carbonation time, d.

### 2.7. Morphology 

Cubic samples with side lengths smaller than 10 mm were cut out from specimens exposed to different conditions with caution to make sure that at least one of the cutting faces contained both aggregates and slurry. These cubic samples were dried at 45 °C in a vacuum drying oven for 7 days. The macro-appearance of aggregates and interface were observed with an industrial electronic microscope at magnification of 50~100 times. The microscopic morphology of ITZ was observed by a scanning electronic microscope (Quanta 250) at an accelerating voltage of 20 kV and current of 20 mA.

### 2.8. Micro-Hardness 

The samples for micro-hardness measurement were prepared in the same way as mentioned in Section 2.7. After drying, these samples were impregnated with epoxy resin for 48 h. The surface of each sample was then polished to easily identify the diamond-shaped indentation. The micro-hardness of the aggregate, ITZ, and matrix zone in both NAC and RAC were then determined by a Vickers indenter. Specifically, the target regions were determined first, then indented within the target regions by applying the load to obtain micro-hardness [44].

## 3. Results and Discussion

### 3.1. Mechanical Properties

(1) Compressive Strength

**Figure 4:**
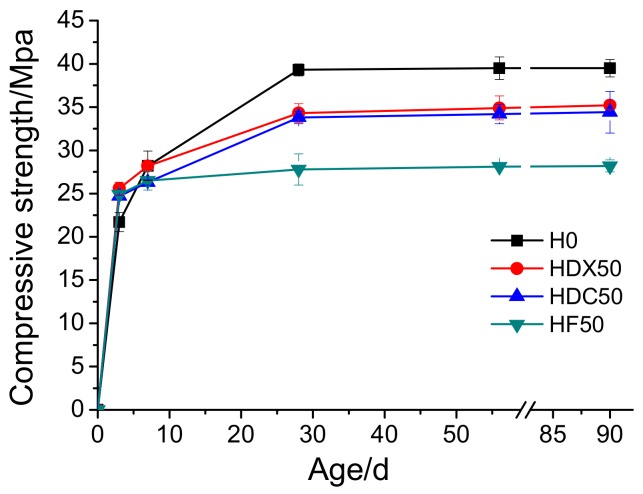
Influence of curing time on the compressive strength of RAC.

Figure 4 shows the impact of curing time on the compressive strength of RAC. One representative mixture of each RAC series was chosen for analysis: HDX50, HDC50, and HF50. It can be observed that the compressive strength of all concrete increased rapidly before 28 days, slowed down afterwards, and stabilized after 56 days. Additionally, the compressive strength of 28 days of all the specimens reached more than 90% of that of 90 days. Moreover, it can also be found that, consistent with the findings of many previous researches [10,11,12,13,14], the incorporation of RA decreased the compressive strength of concrete. Furthermore, the compressive strength reduction of concrete mixed with single RCA was greater than that mixed with single RFA. Furthermore, concrete with both RCA and RFA had the lowest compressive strength, particularly after 10 days of hydration.

**Figure 5:**
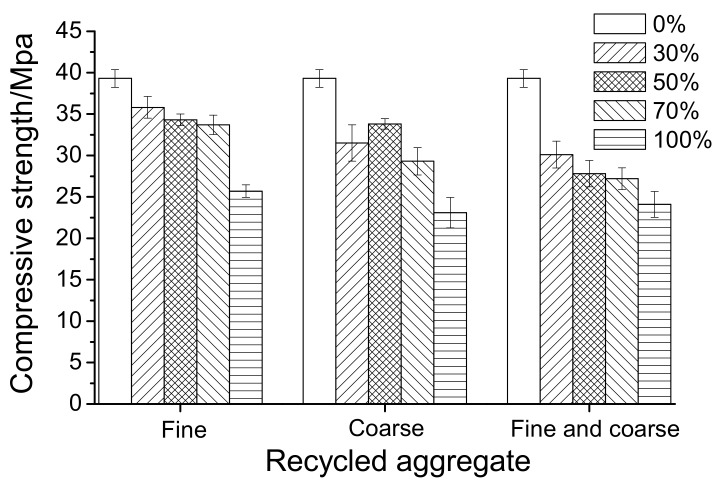
The 28-day compressive strength of RAC with different dosages of recycled aggregates (RA).

Figure 5 presents the compressive strength of RAC with different incorporation of RA at 28 days. It can be observed that the compressive strength of all RAC, either containing only RCA or RFA, or both RCA and RFA, gradually decreased with the increase of RA replacement at 28 days. Compared with natural concrete, RAC with both RCA and RFA had the most significant decrease in compressive strength, then RAC with single RCA, and RAC with single RFA being the least. Though the replacement percentage of the recycle aggregates was the same, the mass incorporated was different. The mass of the recycle aggregates with double replacement of RCA and RFA was the highest, then RCA, and RFA was the least. Since RA has inherent defects, such as numerous micro-cracks, large porosity, and low strength [31,32,37,38], the increase of its content will introduce more flaws into concrete, leading to the rise of fragility and more severe degradation of mechanical performances.

Moreover, it can be seen from Figure 5 that when the RFA dosage was lower than 70%, the compressive strength of specimens was higher than 32.5 MPa and the drop degree was within 15%, which was superior to that of RFA concrete in other studies [9,11,14]. Furthermore, the compressive strength reduced to less than 30 MPa when the dosage of RCA, mixed RCA and RFA, reached 70%, which is significant and coincides with the findings in other studies [7,8,11]. It can also be observed from Figure 5 that the compressive strength of HDC50 did not conform with the regular law of compressive strength, changing with RCA content. This can be attributed to the variations in the composition of RA. The abnormity was acceptable considering the test uncertainties.

(2) Flexural Strength

**Figure 6:**
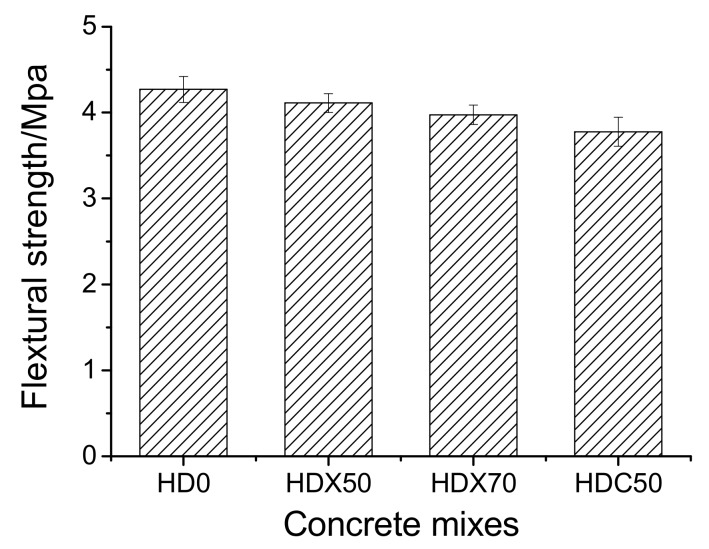
Flexural strength of recycled aggregate concrete at age of 28 days.

Figure 6 shows the flexural strength of RAC at 28 days. It can be seen that the incorporation of RCA weakened the flexural strength of concrete, while the flexural strength of the two RFA mix proportions decreased slightly. The breaking strength of these mixtures was within 3.5~4.5 MPa, which conforms to the previous studies [6,7,8].

**Figure 7:**
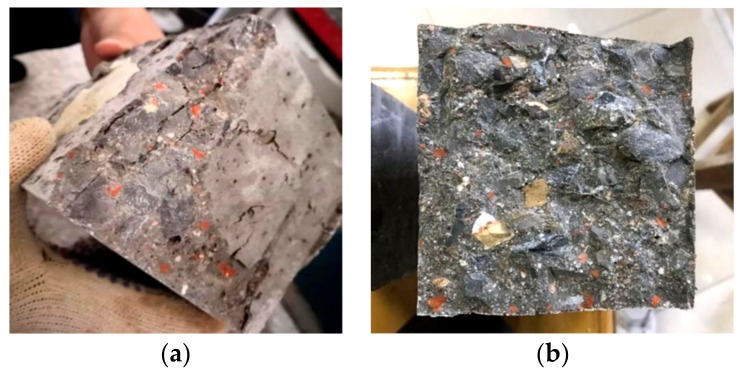
The morphology of fracture surface HDC50: (**a**) after the compression test and (**b**) after the flexural test.

Figure 7 shows the fracture surfaces of RAC specimens. It can be observed that the fractures were mainly caused by three aspects: the interface between RA and paste, the fracture of RA, and the fracture of red brick. They were the major reasons responsible for the poor mechanical performance of RAC.

**Figure 8:**
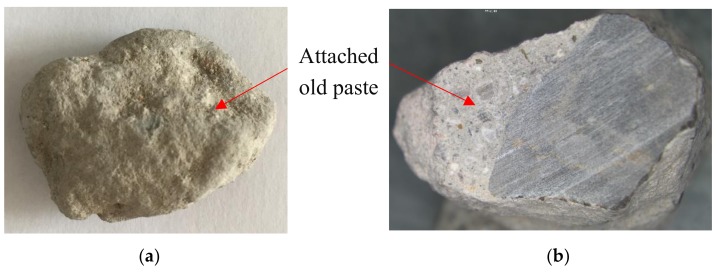
The recycled coarse aggregate attached with old paste: (**a**) appearance; (**b**) cross section.

**Figure 9:**
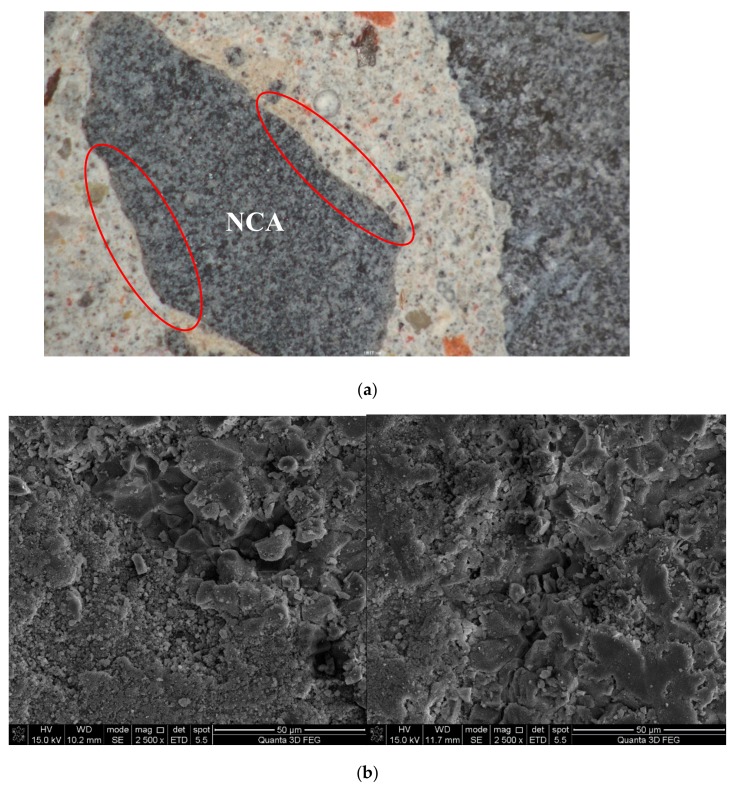
Interface transition zone (ITZ) of natural aggregate concrete (H0): (**a**) mesoscopic morphology; (**b**) microscopic morphology.

**Figure 10:**
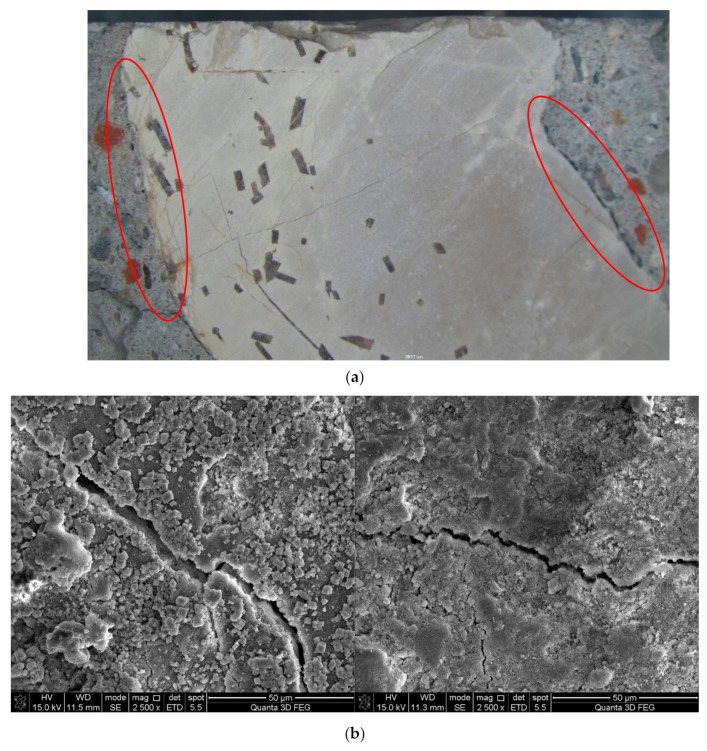
ITZ of recycled aggregate concrete (HDC50): (**a**) meso-morphology (**b**) micro-morphology.

ITZ is the weak region in either NAC or RAC, which is especially worse for RAC because the interface is usually attached with old slurry (see Figure 8). Figure 9 and Figure 10 show the ITZ of NAC and RAC, respectively. It is clear that the ITZ of NAC was denser with no visible cracks or voids; however, there were several cracks between the RA and slurry, which made the ITZ in RAC loose and fragile. Therefore, it can be inferred that that the ITZ was the main reason for the bearing capacity decrease of RAC. This is probably due to that fact that the mass of RCA was higher and the area of ITZ was greater [37,38] and the strength of concrete with RCA was lower than that with RFA, as shown in Figure 5 and Figure 6. Moreover, the slightest strength decline of specimens mixed with only RFA can be ascribed to the RAF itself. RAF mainly consists of broken old pastes and red bricks, and after years of exposure, RFA becomes porous, resulting in high water absorptivity (see Table 2). As a result, cement particles can fill in the pores of RFA and densify the matrix. On the other hand, the water absorbed by RFA in casting can play the role of internal curing [21]. Therefore, the strength of specimens mixed with single RFA was higher.

**Figure 11:**
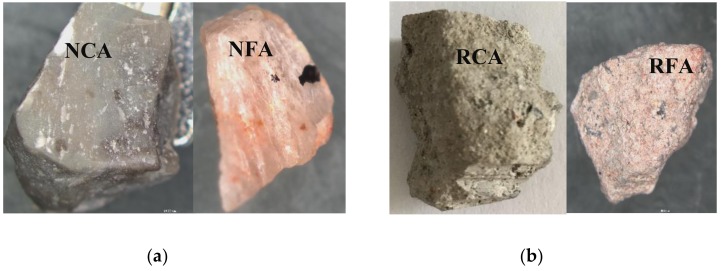
The macro-morphology of natural aggregates and recycled aggregates: (**a**) natural aggregates; (**b**) recycled aggregates.

**Figure 12:**
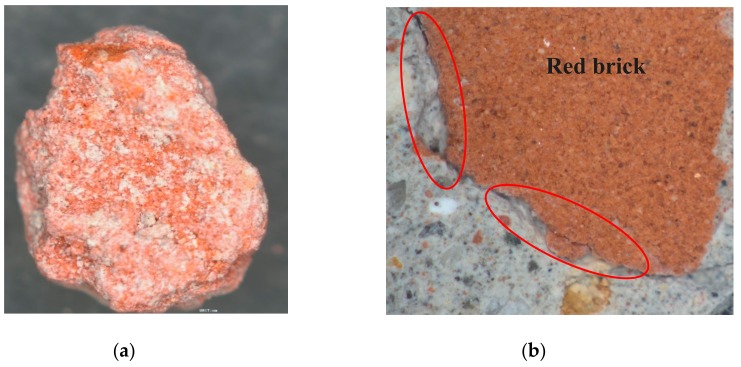
The macro-morphology of (**a**) red block particle and (**b**) meso-morphology of ITZ between the red block particle and paste in RAC.

As for the inherent defects of RA, Figure 11 provides the macro-appearance of NA and RA for comparison. It can be observed that NA has dense and hard texture (Figure 11a) with no visible cracks, whereas RA is porous and loose (Figure 11b) with a number of micro-cracks (Figure 10a). Therefore, the RA breaks easily under force, which decreases the mechanical performances of concrete. Furthermore, the existence of red brick particles also has a negative impact on the mechanical performances of concrete. As shown in Figure 12, red brick is porous and has low strength by itself, and its binding with paste is also weak.

**Figure 13:**
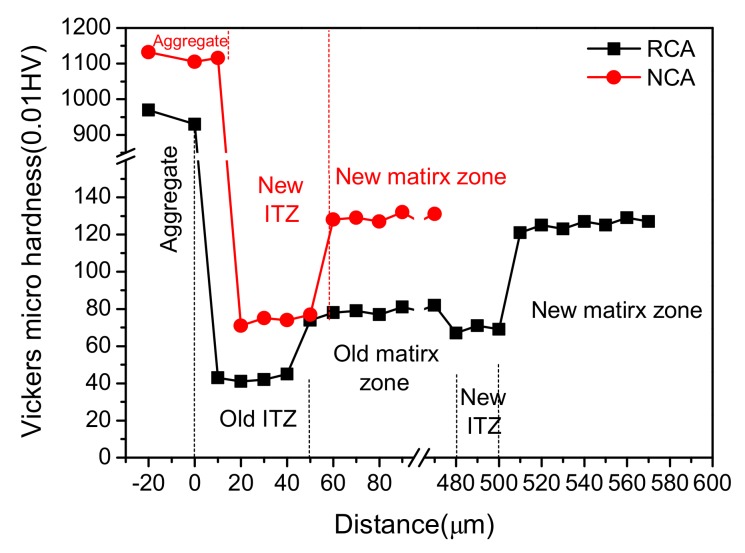
Micro-hardness distribution at the ITZ and matrix zone around recycled coarse aggregate (RCA) and natural coarse aggregate (NCA) in concrete.

Figure 13 presents the Vickers hardness of the matrix zone and ITZ around NCA and RCA in H0 and HDC50. The hardness curve of NCA includes three parts: the coarse aggregate, new ITZ, and new matrix zone, while that of RCA includes five parts: the coarse aggregate, old ITZ, old matrix zone, new ITZ, and new matrix zone. It can be found that the hardness of ITZ was the lowest, then that of the matrix zone, with the hardness of aggregate being the highest, which further demonstrates that ITZ was the weakest region of concrete. Moreover, the hardness of the new matrix zone in RAC was close to that of NAC, and that of new ITZ in RAC was a little smaller than that in NAC. Hence, compared with NAC, the poorer performances of RAC should not be ascribed to the new ITZ and the new matrix zone. Furthermore, it can be seen that the hardness of the old ITZ was the lowest, and the hardness of the old matrix zone was obviously lower than that of new matrix zone in NAC at the same distance from the aggregate. The range of the old matrix zone was wide (about 420 µm from the aggregate for the tested sample), owing to the RCA adopted. Therefore, it can be concluded that the poor performances of RAC was mainly related to the weakness of the old ITZ and the old matrix zone.

### 3.2. Frost Resistance

(1) Process and Characterization of Damage

The appearance changes of RAC and NAC are basically the same with the increase of freezing and thawing cycles. After 25 cycles, the appearance of specimens generally remains intact, and only a small part of RAC specimens have a few edge damages and a small number of voids. After 50 cycles, numerous voids started to appear on the concrete surface, presenting the onset of a pitted surface. The appearance of H0 and HDX50 remained roughly intact, while only a few voids appeared, whereas the surface of HDC50 and HDX70 had a number of voids, and some of them were even connected, leading to a partial pitted surface. After 75 cycles, a pitted surface appeared on the surface of all concrete specimens, and the appearance damage of HDC50 and HDX70 was more severe with large-scale paste spalling. The detailed damage information of the four mix-proportion concrete specimens is presented in Table 5. It can be found that RAC was more severely damaged than NAC, which resulted from the defects of RA itself. Moreover, the damage of RCA concrete was worse than that of RFA concrete. On the one hand, the actual dosage of RCA was greater and there were more defects; on the other hand, the water absorbed by RFA played the role of inner curing and densifying the matrix.

(2) Mass Loss

**Figure 14:**
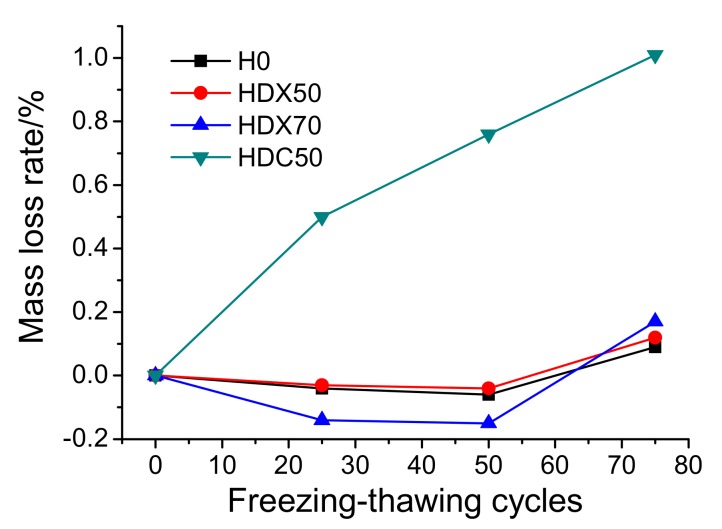
Mass loss rate of concrete specimens after exposed to different freeze-thawing cycles.

Figure 14 shows the mass loss rate of concrete as a function of the freezing and thawing cycles. It can be observed that the mass loss of H0, HDX50, and HDX70 presented a tendency of first decreasing, then increasing with continuous freeze-thawing cycles, which is consistent with the observation of some other researches [45]. It was probably caused by the fact that at the early stage of freezing and thawing, the paste spalling of specimens was slight and the pores in concrete could adsorb a certain amount of water from the external environment, compensating the mass loss to some degree. Especially for the specimens blended with RFA, RFA possessed higher water absorptivity, which could compensate for more mass loss. Therefore, the mass increase of HDX70 in the early stage was the most significant. At the late stage of freezing and thawing, concrete damage was then exacerbated with a large amount of paste or aggregate sapling, leading to the increase of mass loss. As is clearly presented in Figure 14, the mass loss of the three RAC concrete started to take off after 50 freezing and thawing cycles; and at 75 cycles, the mass loss of HDX50 and HDX70 gradually surpassed that of H0. However, the mass loss of HDC50 did not have a decrease phase, and it was probably because HDC50 started paste spalling at 25 freezing and thawing cycles (see Table 5), and hence the mass loss tested after different cycles gradually increased and reached 1.1% at 75 cycles. Consequently, in terms of mass loss within 75 cycles, the frost resistance of RAC with RCA was much worse than that of RAC with RFA, whose frost resistance was only slightly weaker than that of NAC.

(3) Relative Elasticity Modulus

**Figure 15:**
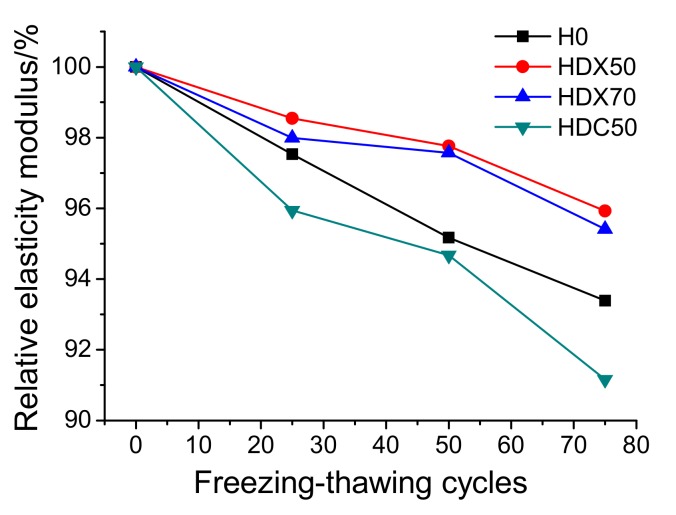
Relative elasticity modulus of concrete specimens after exposed to different freeze-thawing cycles.

Figure 15 presents the relative elasticity modulus change of specimens with freeze-thawing cycles. It is clear that with the increase of freeze-thawing cycles, the relative elasticity modulus of all specimens deceased gradually. Compared with NAC (H0), the relative elasticity modulus of HDX50 and HDX70 were constantly higher, suggesting that RFA promoted the elasticity modulus and thus the frost resistance of concrete. According to previous research [46,47], RAF made up of broken old pastes and red brick particles contains many pores that can provide more space to alleviate the expansion stress during the frost heaving process, and thus has limited negative impact on the relative elasticity modulus of RAC. However, the relative elasticity modulus of HDC50 is significantly lower than that of other mix proportions, and the maximum elasticity modulus loss can reach 8.9%, which means the frost resistance is poorer.

Combining the experiment results of appearance, mass loss, and relative elasticity modulus of specimens, it can be found out that the frost resistance of RAC with RFA (HDX50/HDX70) was not necessarily worse than that of NAC, and its elasticity modulus was even higher. However, the frost resistance of RAC with RCA was significantly poorer than that of NAC. RA itself and ITZ were probably the major factors affecting the frost resistance of RAC.

**Figure 16:**
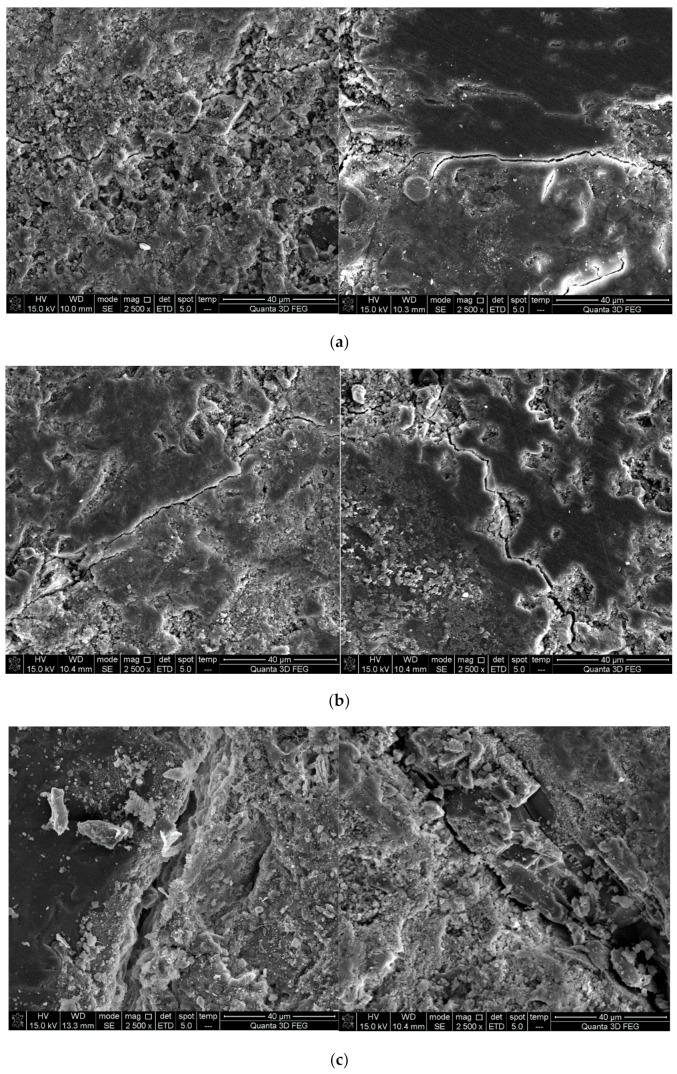
ITZ in H0, HDX50, and HDC50, separately, after freezing and thawing for 50 cycles: (**a**) H0; (**b**) HDX50; (**c**) HDC50.

It can be observed from Figure 11 that RFA had many pores that could alleviate expansion stress. In Figure 10, it is clear that RCA had many fissures that broke easily during frost heaving, leading to the degradation of all the performances of specimens after freeze-thawing cycles. Moreover, Figure 16 shows the ITZ of H0, HDX50, and HDC50 after 50 cycles. It can be observed that the ITZ of HDC50 had more defects than that of H0 and HDX50, which made it more fragile. Therefore, the frost resistance of HDC50 was the worst since it had more inherent defects and no alleviation capability and inner curing effect like RFA. In addition, comparing the ITZ of NAC and RAC before (see Figure 9b and Figure 10b) and after (see Figure 16) subjected to freezing and thawing, it can be found that both the ITZ of NAC and RAC had more defects after freeze-thawing, which was one of the major reasons for the degradation of the concrete.

Based on the above, it can be drawn that the frost resistance of RAC with RFA (HDX50/HDX70) was slightly weaker than that of NAC, while that of RAC with RCA was significantly worse than that of NAC. This is consistent with the findings of Salih et al. [21,22,23], who showed that the relative elastic modulus decreased and the mass loss of concrete increased when exposed to freezing and thawing attack. Moreover, the maximum mass loss and maximum elasticity modulus loss of the three mix-proportion RAC were 1.1% and 8.9%, separately, which met the frost-resistance requirement of the revetment construction of the lower Yellow River (mass loss being no more than 5% and relative elasticity modulus being no less than 60% [42]).

### 3.3. Carbonation Resistance

(1) Carbonation Depth

**Figure 17:**
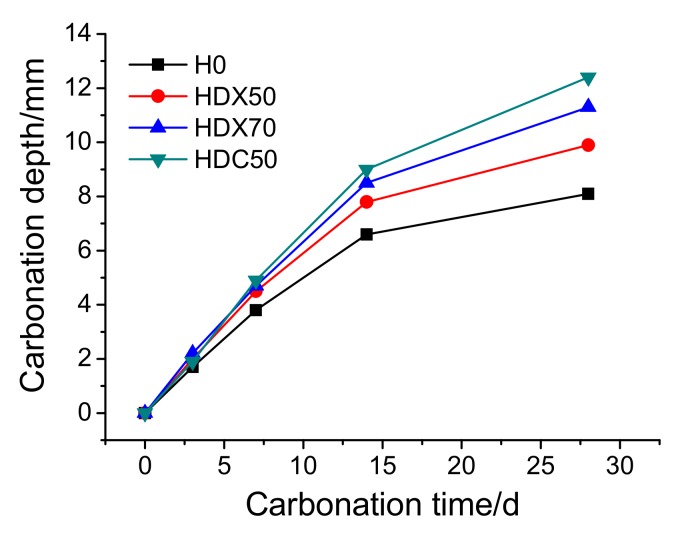
Carbonation depth of concrete specimens at different carbonation time.

The development of carbonation depth of specimens is shown in Figure 17. It can be observed that the carbonation depth of all specimens increased with exposure time. The carbonation depth of RAC was higher than that of NAC, which can be attributed to the larger number of channels for CO_2_ infiltration provided by the micro-cracks and large porosity of RA. The carbonation depth of HDC50 with RCA was the highest, reaching 12.4 mm at 28 days, and that of H0 at 28 days was 7.2 mm, the former of which was 1.7 times higher than the latter, and lower than that the result (2.5 times) of Silva et al. [27]. This demonstrates that RAC in this study had a better carbonation resistance. This is closely related to the actual RA content of RAC; the higher the actual RA content, the more channels for CO_2_ infiltration, leading to a more severe carbonation degree. Moreover, since specimens mixed with RFA possess certain inner curing capacities, the hydration degree was more adequate compared with that of specimens mixed with RCA, and the matrix of RFA was denser, which made CO_2_ infiltration harder. In addition, RFA contained more pastes, which can consume more CO_2_ compared with RCA. These two factors lead to the result that the carbonation depth of RFA concrete was lower than that of RCA concrete.

(2) Carbonation Rate

**Figure 18:**
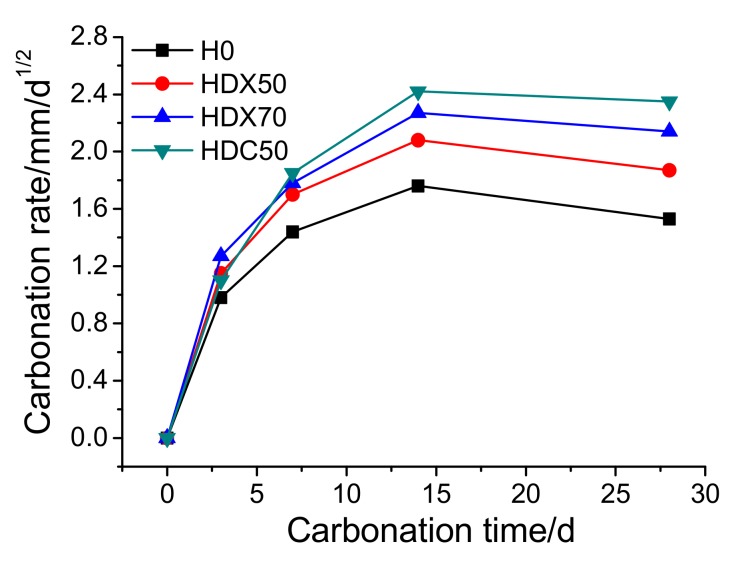
Carbonation rate of concrete specimens at different carbonation times.

**Figure 19:**
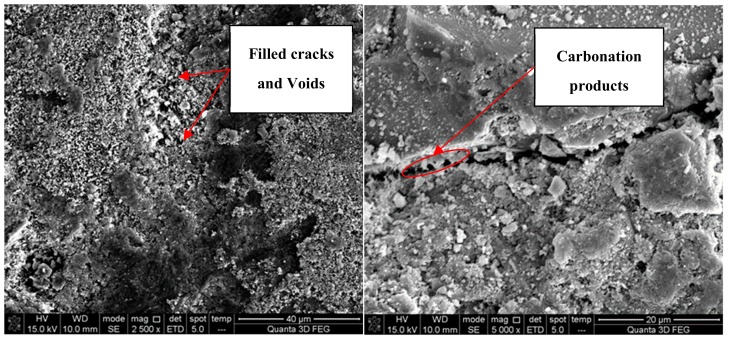
ITZ and micro-morphology of old paste around RCA in RAC (HDC50) after carbonation for 14 days.

**Figure 20:**
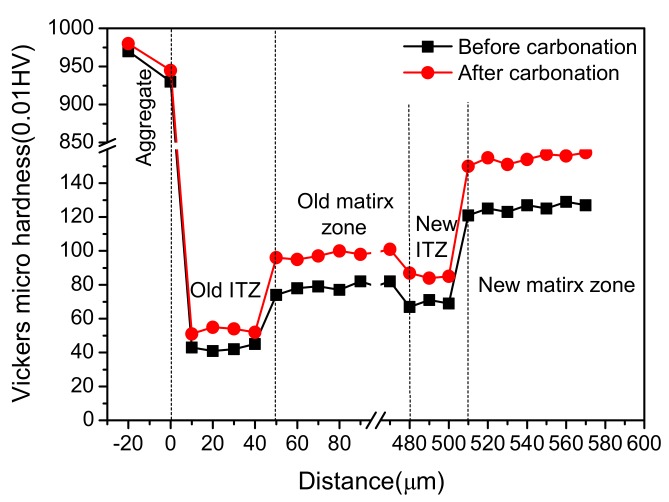
Micro-hardness distribution at the new and old ITZ in RAC before and after carbonation.

Figure 18 presents the carbonation rate of specimens at different carbonation times. It can be found that the carbonation rate of specimens first increased rapidly, then stabilized and decreased slightly. During carbonation, the products insoluble in water gradually formed and filled in the pores in concrete and densifies its microstructure, which cut off the channels for CO_2_ infiltration, mitigating the following carbonation reaction, and thus slowed down the carbonation rate. As is shown in Figure 19, the micro-cracks and voids around ITZ were filled in gradually after carbonation, blocking the further infiltration of CO_2_. Moreover, the Vickers hardness of the ITZ and matrix zone around RA in HDC50 after carbonation is shown in Figure 20. It can be observed that the Vickers hardness of the old ITZ, old matrix zone, new ITZ, and new matrix zone after carbonation all increased significantly, suggesting that the ITZ became stiffer and carbonation improved the performance of RAC. Furthermore, with the distance to RA surface expanding, the increase of Vickers hardness multiplied gradually, revealing that the closer to RA surface, the harder the CO_2_ infiltration, and the weaker the carbonation degree. In addition, the carbonation rate of RAC was significantly higher than that of NAC, with HDC50 being the fastest, followed by HDX70 and HDX50, successively.

According to the above analysis, it can be summarized that the carbonation resistance of RAC was weaker than that of NAC. However, the carbonation depth of RAC specimens at 28 was smaller than 20 mm, which fully met the carbonation-resistance requirement for the revetment construction of the lower Yellow River [42]. Additionally, the mix proportion HDX50 of RAC possessed the best carbonation resistance.

Above all, the chosen mix proportions (HDX50, HDX70, and HDC50) of RAC successfully met the performance requirement of concrete for the revetment construction of the lower Yellow River, in terms of compressive strength, flexural strength, frost resistance, and carbonation resistance. Therefore, the chosen RAC can be applied to the actual revetment engineering of the Yellow river. After analyzing and combining all the experiment results, the engineering application performance of the three mix proportions is ranked as: HDX50 > HDX70 > HDC50. If the engineering application performance and economic performance of material are considered, RAC HDX70 is the best and is recommended to be used in the actual revetment engineering of the lower Yellow River. Moreover, the RAC designed in this study with local raw material possesses superior mechanical properties and durability, which are as good as those in other studies. Therefore, it can be wildly applied, except for the bank revetment engineering of the Yellow River.

## 4. Conclusions

(1) Based on the compressive strength data at 28 days, the RAC of the mix proportions HDX50, HDX70, and HDC50 were selected for compressive strength over 30 Mpa and a RA substitution rate no less than 50%.
(2) Considering the combination of the appearance, mass loss, and relative elasticity modulus situations of specimens after freeze-thawing cycles, HDX50 possessed the best frost resistance performance, followed by HDX70 and HDC50, successively. Improvement on the relative elasticity modulus of HDX50/HDX70 was observed, compared to NAC.
(3) In view of the carbonation depth and rate of RAC, the carbonation resistance of HDX50 was the best, followed by HDX70 and HDC50. Moreover, carbonation could densify the ITZ of RAC and increase its micro-hardness.
(4) RA attached with old pastes had lots of micro-cracks and voids, and the ITZ formed around RA were loose and with low micro-hardness, which were deemed to be the dominating reasons leading to the poorer performance of RAC than that of NAC. In addition, RFA concrete had better performance than RFC concrete, since RFA possessed certain inner curing capacities.
(5) The selected HDX50, HDX70, and HDC50 meet the performance requirement of concrete for the revetment construction of the lower Yellow River in terms of mechanical and durability performance. Combing performance and RA utilization rate, HDX70 ranks first and is recommended to be used in actual revetment engineering.

## Figures and Tables

**Table 1:** The chemical composition of cement (%).

SiO_2_	Al_2_O_3_	Fe_2_O_3_	CaO	MgO	TiO_2_	Na_2_O	K_2_O	SO_3_
21.1	7.81	2.91	54.4	3.187	0.425	0.191	0.399	2.930

**Table 2:** The parameters of recycled coarse and fine aggregates.

Items	Clay (%)	Brick (%)	Elongated and Flaky Particle (%)	Crushing Value (%)	Water Absorption (%)	Apparent Density (kg/m^3^)
RCA	1.1	0.6	6.50	17.6	6.40	2544
RFA	0.7	0.4	/	/	26.3	2562

Note: RCA is recycled coarse aggregate, RFA is recycled fine aggregate.

**Table 3:** The mix proportions of recycled aggregate concrete (RAC).

NO.	W/C	Cement	Coarse Aggregate		Fine Aggregate		Water	Superplasticizer
			Natural	Recycled	Natural	Recycled		
H0	0.39	330	1136	0	760	0	130	1
HDC30	0.39	330	795	341	760	0	130	1 1 1 1
HDC50			568	568				
HDC70			341	795				
HDC100			0	1136				
HDX30	0.39	330	1136	0	532	228	130	1 1 1 2
HDX50					380	380		
HDX70					228	532		
HDX100					0	760		
HF30	0.39	330	795	341	532	228	130	1 2 2 3
HF50			568	568	380	380		
HF70			341	795	228	532		
HF100			0	1136	0	760		

Note: H = revetment, D = single mixing; F = double mixing; C = recycled coarse aggregate; X = recycled fine aggregate, water to cement ratio (W/C).

**Table 4:** Frost resistance requirements for RAC under different service conditions.

Service Conditions		Frost Resistance Grade
Non-heating regions (average temperature in the coldest month >−5 °C)		F15
Heating regions	RH ≤ 50%	F25
	RH > 50%	F35
Positions impacted by cyclic wetting-drying or water level changing		≥F50

Note: Fn means that the minimum number of freeze-thaw cycles that RAC can withstand is n; relative humidity (RH).

**Table 5:** Surface morphology of concrete specimens after different freeze-thaw cycles.

NO.	Cycles		
	25	50	75
H0	Intact	Small number of holes	Large number of holes
HDX50	Intact	Small number of holes	Large number of holes; slight pitted surface
HDX70	Roughly intact	Small number of holes; slight pitted surface	Pitted surface; slight paste spalling
HDC50	Slight damage in corners; Small number of holes	Large number of holes; pitted surface	Pitted surface; paste spalling
